# Efficacy and safety of 40 mg osimertinib administered every other day for non‐small cell lung cancer harboring an epidermal growth factor receptor mutation: A single‐center retrospective cohort study

**DOI:** 10.1111/1759-7714.15226

**Published:** 2024-01-27

**Authors:** Ayumu Otsuki, Naoki Inoshima, Kentaro Tochigi, Hiroyuki Ito, Kei Nakashima

**Affiliations:** ^1^ Department of Pulmonology Kameda Medical Center, Kamogawa Chiba Japan

**Keywords:** epidermal growth factor receptor, non‐small cell lung cancer, weight loss

## Abstract

Osimertinib is a first‐line or adjuvant therapy for non‐small cell lung cancer (NSCLC) harboring an epidermal growth factor receptor (*EGFR*) mutation. However, owing to the adverse events associated with treatment, certain patients cannot maintain a daily regimen of 80 or 40 mg. In this study, we examined the efficacy of 40 mg of osimertinib administered every other day. In this single‐center, retrospective study, we evaluated patients with NSCLC harboring an *EGFR* mutation in whom treatment was initiated with 40 mg osimertinib every other day at our institution between May 2016 and June 2023. The major outcome was the duration of administering 40 mg osimertinib every other day. Six patients with NSCLC were evaluated. The median duration of 40 mg osimertinib intake every other day was 12.6 months. Four of the six patients weighed below 50 kg, and four experienced weight loss. Additionally, four of the six patients had severe renal impairment upon receiving 40 mg osimertinib every other day. Thus, our findings suggest the efficacy of administering 40 mg osimertinib every other day in patients with low bodyweight, weight loss, or severe renal impairment.

## Graphical abstract

In this single‐center, retrospective study, we evaluated patients with non‐small cell lung cancer harboring an epidermal growth factor receptor mutation who were initiated on 40 mg osimertinib every other day. Six patients were evaluated. The median duration of 40 mg osimertinib intake every other day was 12.6 months. Four patients weighed below 50 kg and had severe renal impairment. Thus, this approach could be a treatment option for patients with low bodyweight or severe renal impairment.

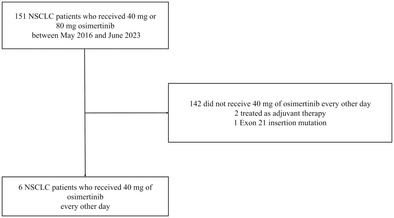

## INTRODUCTION

Epidermal growth factor receptor (*EGFR*) mutations are the most common mutations in non‐small cell lung cancer (NSCLC). Osimertinib is effective against *EGFR* mutation, especially the *EGFR* T790M mutation,^1^ and is recommended as the first‐line therapy for *EGFR*‐mutated NSCLC^2^ and also recommended as adjuvant therapy for completely resected stage II–IIIA *EGFR*‐mutated NSCLC.^3^ According to the FLAURA and ADAURA trials,^2^, ^3^ 15% and 10% of patients, respectively, cannot maintain continuous osimertinib use owing to its adverse effects. Thus, extending the duration of effective osimertinib treatment is essential to maintain activities of daily living (ADL) and prolong survival.

Cytotoxic agents can cause severe adverse events such as nausea and decreased appetite, which are associated with decreased ADL. In phase III trials, the incidence of any grade of nausea and decreased appetite attributable to cytotoxic agents was 33.8%–43.3% and 19.0%–26.0%, respectively.^4^, ^5^, ^6^ Thus, some patients refuse treatment with cytotoxic agents because of their toxicities. The efficacy of gefitinib, a first‐generation *EGFR* tyrosine kinase inhibitor, administered every other day, has been demonstrated.^7^, ^8^ However, the efficacy of 40 mg of osimertinib administered every other day has not yet been reported.

## METHODS

### Study population

In this single‐center, retrospective, cohort study, we included patients who received 40 or 80 mg osimertinib for NSCLC harboring an *EGFR* mutation between May 2016 and June 2023 at the Kameda Medical Center. The exclusion criteria included patients not receiving 40 mg osimertinib every other day, those receiving osimertinib as adjuvant therapy, and those lacking exon 19 deletion or exon 21 L858R point mutation. The Cobas *EGFR* mutation test was used to detect *EGFR* mutations, while PD‐L1 expression was assessed using the PD‐L1 IHC 22C3 pharmDx assay. The study protocol complied with the Declaration of Helsinki and was approved by the Institutional Review Board of Kameda Medical Center “23–032.” The requirement for written informed consent was waived owing to the study's retrospective nature, with no interventions performed on the patients.

### Treatment strategy

All patients received initial osimertinib doses of 80 mg. Doses were tapered to 40 mg once daily, followed by 40 mg every other day when patients could not be reliably administered a specific dose owing to adverse events. The attending physician decided the initial dose of osimertinib and reduced it according to adverse events experienced.

### Data collection

Data regarding patient age, sex, performance status, height, bodyweight, body mass index, pathology of lung cancer, TNM stage (according to the staging criteria of the American Joint Committee on Cancer, eighth edition), *EGFR* mutation status, total proportion of score of PD‐L1 (TPS), treatment line, blood test (to detect white blood cell and platelet counts and aspartate aminotransferase, alkaline phosphatase, creatine phosphatase, albumin, and creatinine levels), duration of osimertinib use at any dose, best response to initial treatment, and details of each adverse event were extracted from medical records. TPS was classified into three groups (high, ≥50%; low, 1–49%: negative, <1%).

The major outcome was the duration of administering 40 mg osimertinib every other day. The total duration of osimertinib administration at any dose, the progression‐free survival (PFS) for patients receiving 40 mg of osimertinib every other day, and relative dose intensity (RDI) of the whole duration of administering osimertinib were assessed. PFS was defined as the time from the initiation of osimertinib to disease progression or death from any cause. Efficacy was assessed from the computed tomography results according to the Response Evaluation Criteria in Solid Tumors version 1.1. Adverse events were graded using the National Cancer Institute Common Terminology Criteria for Adverse Events version 5.0. Categorical variables are shown as percentages (%), and continuous variables as medians (25–75 percentiles). The Kaplan–Meier method was used to estimate the dose duration and PFS for patients taking 40 mg osimertinib every other day and the overall duration of osimertinib administration at any dose using EZR on R commander version 1.61. This was a retrospective case study with a limited number of patients; thus, we did not use hypothetical testing.

## RESULTS

**FIGURE 1:**
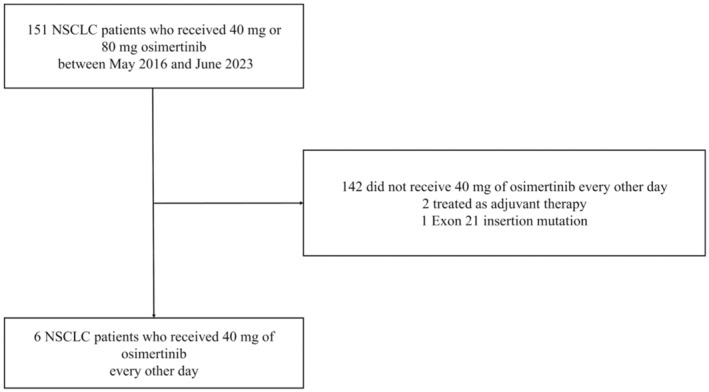
Patient disposition in this study. NSCLC, non‐small cell lung cancer.

Of the 151 patients receiving osimertinib at varying doses and treatment lines for NSCLC harboring *EGFR* mutations, six patients met the inclusion criteria (Figure 1). The median age was 79 years, and five were female. All patients' performance statuses were 0 or 1. All histological findings confirmed adenocarcinomas, and five of the six cases harbored the L858R mutation. Four of the six patients had severe renal impairment (Table 1). The median duration of 40 mg osimertinib administered every other day and median PFS were both 12.6 months (3.0–NA), the median overall duration of osimertinib treatment was 30.7 months (17.5–NA), and the median RDI was 45.1%. As of June 30, 2023, two patients received 40 mg osimertinib every other day (Table 2). Four patients ceased osimertinib treatment because of tumor progression. Four patients maintained their performance status, whereas two patients with worsened performance opted not to receive second‐line therapy. Of the six, four patients' bodyweights were below 50 kg, and four patients lost weight compared with that before taking the initial dose of osimertinib and at the start of 40 mg osimertinib administration every other day. Furthermore, five patients experienced renal impairment, and two patients' renal impairment worsened before taking 40 mg osimertinib every other day. In one patient, grade 3 platelet count decreased and creatine phosphokinase levels increased. Two patients experienced appetite loss and an acneiform rash (Table 3).

**TABLE 1:** Patient characteristics.

Patient characteristics	Overall population
Median age (IQR)	79 (78–81)
Female, *n* (%)	5 (83)
Smoking history *n* (%)	0 (0)
Performance status, *n* (%)	
0	3 (50)
1	3 (50)
Histology, *n* (%)	
Adenocarcinoma	6 (100)
*EGFR* mutation status, *n* (%)	
L858R	5 (83)
Ex19del	1 (17)
Total proportion score of PD‐L1, *n* (%)	
Low	4 (66)
Negative	2 (34)
Treatment line, *n* (%)	
First	4 (66)
Second	1 (17)
Third	1 (17)
Before osimertinib treatment	
Body mass index (kg/m^2^) (IQR)	23.2 (20.7–25.4)
Blood test, median (IQR)	
White blood cell (/μL)	5600 (4800–6600)
Platelet (×10^4^/μL)	21.8 (14.0–22.4)
Aspartate aminotransferase (U/L)	24.0 (21.5–28.0)
Alkaline phosphatase (U/L)	17.0 (14.5–21.8)
Albumin (g/dL)	3.8 (3.50–3.89)
Creatinine clearance (mL/min)	48.5 (33.9–50.2)
Before osimertinib 40 mg every other day	
Body mass index (kg/m^2^) (IQR)	21.5 (19.8–22.1)
Blood test, median (IQR)	
White blood cell (/μL)	5500 (4400–6200)
Platelet (×10^4^/μL)	19.2 (11.8–23.4)
Aspartate aminotransferase (U/L)	24.0 (22.2–29.5)
Alkaline phosphatase (U/L)	18.5 (13.8–23.2)
Albumin (g/dL)	3.8 (3.5–4.0)
Creatinine clearance (mL/min)	34.9 (29.4–43.3)

Abbreviation: IQR, interquartile range.

**TABLE 2:** Results of this study.

Median whole duration of osimertinib (months) (95% CI)	30.7 (17.5–NA)
Median duration of osimertinib 40 mg every other day (months) (95% CI)	12.6 (3.0–NA)
Median progression‐free survival (months) (95% CI)	12.6 (3.0–NA)
Median relative dose intensity (%) (IQR)	45.1 (40.9–50.3)
Number of continuations of taking osimertinib, *n* (%)	2 (33.3)

Abbreviations: CI, confidence interval; IQR, interquartile range.

**TABLE 3:** Characteristics of the six patients.

Patient number	1	2	3	4	5	6
Age (year)	79	82	81	76	77	79
Sex	Female	Male	Female	Female	Female	Female
Smoking history	None	None	None	None	None	None
Performance status	1	1	0	0	1	0
Pathology	Ad	Ad	Ad	Ad	Ad	Ad
TNM stage (eighth)	IVA	IVB	IVA	Recurrence	IIB	IVB
Mutation status	L858R	L858R	Ex19del	L858R	L858R	L858R
TPS	Low	Low	Low	Low	Negative	Negative
Line	First	First	Third	Second	First	First
Before taking the initial dose osimertinib						
Height (cm)	148.2	158.8	142.0	146.0	148.0	137.6
Bodyweight (kg)	58.7	60.0	45.0	42.8	37.2	48.5
Body mass index (kg/m^2^)	26.8	24.0	22.3	20.1	17.0	25.8
WBC (/μL)	8800	6800	5200	3900	4700	5900
Platelet (×10^4^/μL)	23.4	22.5	21.4	11.6	10.2	22.1
AST (U/L)	25	17	21	35	23	29
ALT (U/L)	14	12	16	24	18	23
Albumin (g/dL)	3.8	3.3	3.4	3.8	4.2	3.9
Creatinine clearance (mL/min)	62.2	49.3	50.6	29.3	47.7	22.1
Before taking osimertinib 40 mg every other day						
Bodyweight (kg)	50.8	53.5	45.0	40.9	38.2	40.5
Body mass index (kg/m^2^)	23.2	21.4	22.3	19.2	17.4	21.6
WBC (/μL)	9400	6000	4200	6300	3300	5000
Platelet (×10^4^/μL)	23.2	23.5	15.1	10.6	6.1	26.3
AST (U/L)	22	40	25	23	31	22
ALT (U/L)	11	40	24	16	21	13
Albumin (g/dL)	3.7	3.3	3.8	3.4	4.2	4.1
Creatinine clearance (mL/min)	34.1	35.6	45.9	24.4	47.4	27.8
Best response to initial treatment	Partial response	Stable disease	Partial response	Partial response	Partial response	Stable disease
Caused by dose reduction						
80 mg osimertinib	G2 dysgeusia	G1 diarrhea	G2 appetite loss	G1 AST, ALT increased	G3 platelet count decreased	G3 CPK increased
40 mg osimertinib	G2 mucositis oral	G2 appetite loss	G2 rash acneiform	G3 QT corrected interval prolonged	G2 platelet count decreased	G2 rash acneiform
Adverse events after dose reduction	Improved (G1)	Improved (G1)	Improved (G1)	Improved (G1)	Not changed	Improved (G1)
Results of this study						
Whole duration of osimertinib (months)	52.6	20.5	49.2	37.1	17.5	24.3
Duration of osimertinib 40 mg every other day (months)	16.8	4.8	41.0	12.9	12.2	3.0
Progression‐free survival (months)	16.8	4.8	41.0	12.9	12.2	3.0
Relative dose intensity (%)	48.0	51.1	30.4	42.2	40.4	62.7
Continuation or end of taking osimertinib	Continuation	End	Continuation	End	End	End
Performance status when osimertinib terminated		1		0	3	2

Abbreviations: ALT, alanine aminotransferase; AST, aspartate aminotransferase; CPK, creatine phosphokinase; TPS, total proportion score of PD‐L1; WBC, white blood cell.

## DISCUSSION

According to the AURA trial,^9^ the objective response rate associated with 20 mg osimertinib once daily was 52%. These data showed that 40 mg osimertinib administered every other day could benefit some patients. The mean (min–max) area under the concentration‐time curve from 0 to time τ over the dosing interval at steady state (AUCτ) for 20 mg and 40 mg of osimertinib was 1965 (873–4990) and 5636 (2040–14 100) nMh, respectively, and the mean (min–max) Cmax values were 106.3 (45.4–280) nM and 305.1 (128–807) nM, respectively. This finding suggests that the AUCτ and Cmax for a dose of 20 mg is expected to be comparable to those for a dose of 40 mg for some patients. In this study, the plasma concentration of 40 mg osimertinib administered every other day might be similar to that of 40 mg osimertinib administered once daily in regular patients because the change in their bodyweight and renal function might influence the pharmacokinetic (PK) model of osimertinib. Osimertinib dose reduction has been reported as a risk to brain metastasis control.^10^ Consequently, it is necessary to carefully consider the risks associated with osimertinib dose reduction. No patient had brain metastasis after taking osimertinib 40 mg every other day in this study.

Bodyweight has the largest effect on the PK model.^11^ Moreover, toxicity occurs more frequently in patients with moderate renal impairment and low bodyweight,^12^ and osimertinib's PK may be related to ethnicity and food.^13^ Here, three of the six patients lost over 5 kg bodyweight, and four of the six patients' bodyweights were under 50 kg when they started osimertinib administration every other day. Inability of such patients to tolerate osimertinib may be attributed to their low bodyweight.^11^, ^12^ After the dose de‐escalation of osimertinib, the patients continued osimertinib treatment. Patients with severe renal impairments reportedly have 1.85‐ and 1.62‐fold higher exposure to osimertinib and its metabolites, respectively, than patients with normal renal function.^14^ Four out of six patients had severe renal impairment when they started receiving osimertinib every other day. Here, plasma osimertinib concentrations might have been high in some patients owing to renal impairment. According to the FLAURA study,^2^ interstitial lung disease, pneumonitis, and QT prolongation are the most common events leading to permanent discontinuation in the osimertinib arm. Plasma osimertinib concentration can affect the probability of rash and diarrhea.^12^ In this study, patients experienced rash, diarrhea, appetite loss, and QT prolongation. However, these adverse events resolved after dose de‐escalation of osimertinib and patients could continue osimertinib treatment. Therefore, we should be careful of the osimertinib dose in a patient with low bodyweight or severe renal failure.^11^

Our study had some limitations. It was retrospective in nature, and there were variations in decision‐making regarding osimertinib dose changes. Additionally, a limited number of participants were included in this study, and blood osimertinib concentrations were not determined.

In conclusion, our findings suggest the efficacy of administering 40 mg osimertinib every other day in patients with *EGFR*‐mutant NSCLC exhibiting low bodyweight, weight loss, or severe renal impairment, particularly when standard‐dose osimertinib is not well‐tolerated.

## AUTHOR CONTRIBUTIONS

All authors had full access to the data in the study and take responsibility for the integrity of the data and the accuracy of the data analysis. Ayumi Otsuki: Conceptualization. Kei Nakashima: Methodology. Ayumi Otsuki and Kentaro Tochigi: Investigation. Kei Nakashima: Formal analysis. Kentaro Tochigi: Resources. Ayumu Otsuki: Writing–original draft. Ayumi Otsuki, Naoki Inoshima, Kentaro Tochigi, Hiroyuki Ito and Kei Nakashima: Writing–review and editing. Hiroyuki Ito: Validation. Kei Nakashima: Supervision.

## CONFLICT OF INTEREST STATEMENT

The authors declare no conflicts of interest.
